# Evaluating the influence of prompt formulation on the reliability and repeatability of ChatGPT in implant-supported prostheses

**DOI:** 10.1371/journal.pone.0323086

**Published:** 2025-05-30

**Authors:** Yolanda Freire, Andrea Santamaría Laorden, Jaime Orejas Pérez, Ignacio Ortiz Collado, Margarita Gómez Sánchez, Israel J. Thuissard Vasallo, Víctor Díaz-Flores García, Ana Suárez

**Affiliations:** 1 Department of Preclinical Dentistry II, Faculty of Biomedical and Health Sciences, Universidad Europea de Madrid, Villaviciosa de Odón, Madrid, Spain; 2 Department of Preclinical Dentistry I. Faculty of Biomedical and Health Sciences, Universidad Europea de Madrid, Villaviciosa de Odón, Madrid, Spain; 3 School for Doctoral Studies and Research. Universidad Europea de Madrid, Villaviciosa de Odón, Madrid, Spain; Dental Hypothesis, IRAN, ISLAMIC REPUBLIC OF

## Abstract

Language models (LLMs) such as ChatGPT are widely available to any dental professional. However, there is limited evidence to evaluate the reliability and reproducibility of ChatGPT-4 in relation to implant-supported prostheses, as well as the impact of prompt design on its responses. This constrains understanding of its application within this specific area of dentistry. The purpose of this study was to evaluate the performance of ChatGPT-4 in generating answers about implant-supported prostheses using different prompts. Thirty questions on implant-supported and implant-retained prostheses were posed, with 30 answers generated per question using general and specific prompts, totaling 1800 answers. Experts assessed reliability (agreement with expert grading) and repeatability (response consistency) using a 3-point Likert scale. General prompts achieved 70.89% reliability, with repeatability ranging from moderate to almost perfect. Specific prompts showed higher performance, with 78.8% reliability and substantial to almost perfect repeatability. The specific prompt significantly improved reliability compared to the general prompt. Despite these promising results, ChatGPT’s ability to generate reliable answers on implant-supported prostheses remains limited, highlighting the need for professional oversight. Using specific prompts can enhance its performance. The use of a specific prompt might improve the answer generation performance of ChatGPT.

## Introduction

Large Language Models (LLMs) are a category of artificial intelligence (AI) specifically designed to emulate human language processing capabilities [1]. These models have been developed through extensive training on massive databases. They are characterized by their strong ability to contextualize and interpret human language [2], which allows them to generate human-like responses [3].

In the field of LLMs, the Generative Pre-trained Transformers (GPT) series from OpenAI [4] have gained prominence as one of the most advanced implementations models. ChatGPT-3.5 was introduced in November 2022n [5] and ChatGPT-4 was introduced in March 2023 [6]. Currently, ChatGPT has positioned itself as one of the most comprehensive and accessible language models to the public [7], widely used by millions of users for various purposes [4], including the search for health-related information [2]. In dentistry, several studies have evaluated the performance of ChatGPT in areas such as Endodontics [8,9], Oral and Maxillofacial Surgery [10,11], Periodontics [12,13], Orthodontics [14–16] or Prosthodontics [17]. In prosthodontics, ChatGPT was found to have limited ability to generate answers related to removable dental prostheses and tooth-supported fixed dental prostheses [17]. However, to date, the performance in implant-supported prosthesis is limited. This area is particularly relevant due to the complexity of implant prosthodontics, which requires the integration of biomechanical principles, prosthetic design, and peri-implant health considerations [18–21]. Unlike other fields of dentistry, where treatment protocols may be more standardized, implant-supported restorations demand highly individualized decision-making based on patient-specific factors [22]. Given that AI tools like ChatGPT are increasingly used for educational and clinical support, assessing their reliability in this domain is essential to determine their potential usefulness and limitations.

In addition, when using these models, possible response bias should be considered. Among the most significant biases is the possibility of producing meaningless content [23] by presenting incorrect information as if it were accurate [24]. The phenomenon where the model generates answers that appear reliable but lack substance or relevance has been described as artificial hallucination [25]. Another possible bias could be related to the use of prompts, as these models have the ability to capture the nuances and complexities of human language through input prompts [7]. Therefore, the generation of answers by ChatGPT depends on prompts entered by users [4]. In this context, prompt engineering is becoming increasingly important, focusing on the design, improvement, and implementation of these prompts to guide the results of LLMs towards concrete answers, thus optimising the interaction with artificial intelligence systems. However, there is a lack of studies analysing the influence of prompts on the answers obtained [26].

Therefore, given the lack of studies in implant-supported prosthesis, it is important to evaluate the performance of ChatGPT to determine its reliability and reproducibility, as well as its performance depending on the type of prompt used, to provide a critical insight into a potential use.

Thus, the aim of this study was to analyze the reliability and repeatability of ChatGPT-4 answer generation to specific implant-supported and implant-retained prostheses questions, and to compare different prompts in generating the answers.

The research hypothesis was that the implant prosthetics answers provided by ChatGPT-4 would not exhibit reliability and repeatability, and that there would be no significant differences between the use of different prompts.

## Materials and methods

This research adhered to the Declaration of Helsinki and did not require ethical approval as it did not involve human participants.

The methodology of this study was based on previous studies published in the literature [8,10,17], adapted to the objectives of this research. The STAGER: Standardized Testing and Assessment Guidelines for Evaluating Generative Artificial Intelligence Reliability [27] and the TRIPOD [28] checklists were used to guide the reporting of this study (S1 and S2 Appendix).

Two authors (A.S., Y.F.) with experience in the design of questions for answer generation in ChatGPT-4 [8,10,17], developed an initial set of 60 questions related to implant-supported and implant-retained prostheses. The questions were designed based on clinical practice guidelines, specifically The Proceedings of the Seventh ITI Consensus Conference of the International Team for Implantology (ITI) [29]. These guidelines were selected as a reference to ensure that the questions covered key aspects of implant-supported prostheses in a structured and evidence-based manner.

An expert judgement approach was used to assess the readability and clinical relevance of the answers generated by ChatGPT-4. These questions were independently evaluated by 2 prosthodontic graduate program faculty members (I.O.C, J.O.P.) for clarity, relevance, and inclusion of key concepts, using a 3-point Likert scale (0 = disagree; 1 = neutral; 2 = agree). To minimize selection bias, the evaluation was blinded to the study objectives. Discrepancies in this evaluation were reviewed by a third prosthodontic graduate program faculty member (A.S.L.). Based on the evaluation scores, the 30 highest-rated questions were selected to ensure a representative and unbiased assessment of ChatGPT’s performance.

To compare the performance of ChatGPT-4 according to the type of prompt, 2 question formats were designed, one general and one specific. The general prompt consisted of a question with no additional instructions [9,11]. The specific prompt was characterized by being more specific and direct, aiming to guide ChatGPT towards more relevant answers [10] Therefore, ChatGPT was instructed to assume the role of a prosthodontist and the target audience was a general dentist, and to answer the questions accurately and directly, without digressions or creative answers. The selected prompt was ‘Imagine that you are a prosthodontist and I am a general dentist. Please answer the following question accurately and directly, without rambling or creative answers.’

Two authors (M.G.S., V.DF.G.), independently and using 2 different ChatGPT-4 Plus accounts, entered the 30 previously selected questions using the 2 types of prompts (Tables 1 and 2). As a result, 60 answers were generated, 30 for each type of prompt. In order to assess the repeatability, 30 answers were obtained for each of the questions. This process was repeated 3 times during the day (morning, afternoon, and evening) in March 2024, using the “new chat” option for each question to reduce memory retention bias [30,31].

**Table 1:** Questions included for ChatGPT-4 to generate answers using the general prompt.

Question Number	Description of the question introduced in ChatGPT with the general prompt
1	What are the disadvantages of manufacturing methods for implant-supported restorations?
2	What are the advantages of additive manufacturing techniques for implant-supported restorations?
3	What are the consequences of poor of marginal fit in implant fixed partial dentures?
4	What are the components of the titanium base abutment geometry?
5	Why do cemented crowns have a higher risk of peri-implant disease than screw-retained restorations?
6	Regarding the emergence profile of an implant-abutment prosthesis complex, which material has reduced plaque retention and demonstrates a better quality of soft tissue attachment?
7	What has been the main technical problem with veneered zirconia implant-supported restorations?
8	What is the most commonly reported complication of titanium base abutments?
9	What factors contribute to the retention of suprastructures on titanium base abutments?
10	How many implants are needed to support a fixed restoration to replace three missing teeth in the posterior region?
11	What is the restorative material of choice for posterior multi-unit fixed implant-supported restorations?
12	What are the advantages of implant-retained overdentures as compared to conventional removable complete dentures?
13	In terms of patient-reported dental outcomes, what is the optimal number of implants to retain a mandibular implant overdenture?
14	What type of prosthesis improves chewing in edentulous patients with an opposing maxillary complete denture?
15	What are the challenges of implant-supported fixed prosthesis?
16	What mechanical factors determine whether a prosthesis can withstand the physiological occlusal forces?
17	What determines the precision of the stereolithography method?
18	What determines the precision of the digital light processing method?
19	What are the advantages of Titanium Base abutments for implant prostheses?
20	What are the adverse effects of increasing the translucency of zirconia?
21	In terms of annual ceramic fracture and chipping rates, do monolithic or veneered implant-supported multiunit restorations perform better in the posterior area?
22	When restoring an edentulous mandible with an implant overdenture, do bars or single attachments provide a better improvement in patient-reported dental outcomes?
23	Between metals and acrylics, which material gives better results in additive manufacturing techniques in iFPDs?
24	What is the main difference between stereolithography and digital light processing?
25	What is the minimum number of implants required for a full-arch fixed implant-supported denture?
26	What should be the shoulder height of the titanium base abutment for bone level conical-connection implants?
27	What type of implant restorations are 3D resins safe for?
28	Which type of attachment is associated with higher satisfaction in implant overdenture patients?
29	How can masticatory performance be objectively assessed?
30	Based on dental patient-reported outcomes, which type of full arch implant prosthesis provides the highest level of stability, retention and comfort?

**Table 2:** Questions included for ChatGPT-4 to generate answers using the specific prompt.

Question Number	Description of the question introduced in ChatGPT with the specific prompt
1	Imagine that you are a prosthodontist and I am a general dentist. Please answer the following question accurately and directly, without rambling or creative answers: What are the disadvantages of subtractive manufacturing methods for implant-supported restorations?
2	Imagine that you are a prosthodontist and I am a general dentist. Please answer the following question accurately and directly, without rambling or creative answers: What are the advantages of additive manufacturing techniques for implant-supported restorations?
3	Imagine that you are a prosthodontist and I am a general dentist. Please answer the following question accurately and directly, without rambling or creative answers: What are the consequences of poor marginal fit in implant fixed partial dentures?
4	Imagine that you are a prosthodontist and I am a general dentist. Please answer the following question accurately and directly, without rambling or creative answers: What are the components of the titanium base abutment geometry?
5	Imagine that you are a prosthodontist and I am a general dentist. Please answer the following question accurately and directly, without rambling or creative answers: Why do cemented crowns have a higher risk of peri-implant disease than screw-retained restorations?
6	Imagine that you are a prosthodontist and I am a general dentist. Please answer the following question accurately and directly, without rambling or creative answers: Regarding the emergence profile of an implant-abutment prosthesis complex, which material has reduced plaque retention and demonstrates a better quality of soft tissue attachment?
7	Imagine that you are a prosthodontist and I am a general dentist. Please answer the following question accurately and directly, without rambling or creative answers: What has been the main technical problem with veneered zirconia implant-supported restorations?
8	Imagine that you are a prosthodontist and I am a general dentist. Please answer the following question accurately and directly, without rambling or creative answers: What is the most commonly reported complication of titanium base abutments?
9	Imagine that you are a prosthodontist and I am a general dentist. Please answer the following question accurately and directly, without rambling or creative answers: What factors contribute to the retention of suprastructures on titanium base abutments?
10	Imagine that you are a prosthodontist and I am a general dentist. Please answer the following question accurately and directly, without rambling or creative answers: How many implants do you need to support a fixed restoration to replace at least three missing teeth in the posterior region?
11	Imagine that you are a prosthodontist and I am a general dentist. Please answer the following question accurately and directly, without rambling or creative answers: What is the restorative material of choice for posterior multi-unit fixed implant-supported restorations?
12	Imagine that you are a prosthodontist and I am a general dentist. Please answer the following question accurately and directly, without rambling or creative answers: What are the advantages of implant-supported overdentures as compared to conventional removable complete dentures?
13	Imagine that you are a prosthodontist and I am a general dentist. Please answer the following question accurately and directly, without rambling or creative answers: In terms of patient-reported dental outcomes, what is the optimal number of implants to retain a mandibular implant overdenture?
14	Imagine that you are a prosthodontist and I am a general dentist. Please answer the following question accurately and directly, without rambling or creative answers: What type of prosthesis improves chewing in edentulous patients with an opposing maxillary complete denture?
15	Imagine that you are a prosthodontist and I am a general dentist. Please answer the following question accurately and directly, without rambling or creative answers: What are the challenges of implant-supported fixed prosthesis?
16	Imagine that you are a prosthodontist and I am a general dentist. Please answer the following question accurately and directly, without rambling or creative answers: What mechanical factors determine whether a prosthesis can withstand the physiological occlusal forces?
17	Imagine that you are a prosthodontist and I am a general dentist. Please answer the following question accurately and directly, without rambling or creative answers: What determines the precision of the stereolithography method?
18	Imagine that you are a prosthodontist and I am a general dentist. Please answer the following question accurately and directly, without rambling or creative answers: What determines the precision of the digital light processing method?
19	Imagine that you are a prosthodontist and I am a general dentist. Please answer the following question accurately and directly, without rambling or creative answers: What are the advantages of Titanium Base abutments for implant prostheses?
20	Imagine that you are a prosthodontist and I am a general dentist. Please answer the following question accurately and directly, without rambling or creative answers: What are the adverse effects of increasing the translucency of zirconia?
21	Imagine that you are a prosthodontist and I am a general dentist. Please answer the following question accurately and directly, without rambling or creative answers: In terms of annual ceramic fracture and chipping rates, do monolithic or veneered implant-supported multiunit restorations perform better in the posterior area?
22	Imagine that you are a prosthodontist and I am a general dentist. Please answer the following question accurately and directly, without rambling or creative answers: When restoring an edentulous mandible with an implant overdenture, do bars or single attachments provide a better improvement in patient-reported dental outcomes?
23	Imagine that you are a prosthodontist and I am a general dentist. Please answer the following question accurately and directly, without rambling or creative answers: Between metals and acrylics, which material gives better results in additive manufacturing techniques in iFPDs?
24	Imagine that you are a prosthodontist and I am a general dentist. Please answer the following question accurately and directly, without rambling or creative answers: What is the main difference between stereolithography and digital light processing?
25	Imagine that you are a prosthodontist and I am a general dentist. Please answer the following question accurately and directly, without rambling or creative answers: What is the minimum number of implants required for a full-arch fixed implant-supported denture?
26	Imagine that you are a prosthodontist and I am a general dentist. Please answer the following question accurately and directly, without rambling or creative answers: What should be the shoulder height of the titanium base abutment for bone level conical-connection implants?
27	Imagine that you are a prosthodontist and I am a general dentist. Please answer the following question accurately and directly, without rambling or creative answers: What type of implant restorations are 3D resins safe for?
28	Imagine that you are a prosthodontist and I am a general dentist. Please answer the following question accurately and directly, without rambling or creative answers: Which type of attachment is associated with higher satisfaction in implant overdenture patients??
29	Imagine that you are a prosthodontist and I am a general dentist. Please answer the following question accurately and directly, without rambling or creative answers: How can masticatory performance be objectively assessed?
30	Imagine that you are a prosthodontist and I am a general dentist. Please answer the following question accurately and directly, without rambling or creative answers: Based on dental patient-reported outcomes, which type of full-arch implant prosthesis provides the highest level of stability, retention and comfort?

The 1800 answers generated by ChatGPT-4 were independently evaluated by 2 prosthodontic graduate program faculty members (expert 1, J.O.P.; expert 2, I.O.C) who were blinded to the study objectives. A 3-point Likert scale was used for assessment (Table 3). Discrepancies in the evaluation were resolved by a third prosthodontic graduate program faculty member (expert 3, A.S.L.). The experts had 3 years of experience in natural language processing and artificial intelligence in this field.

**Table 3:** Grading system for answers generated by ChatGPT.

Grading	Grading description
Incorrect (0)	The answer provided is completely incorrect or unrelated to the question. It does not demonstrate an adequate understanding or knowledge of the topic.
Partially correct or incomplete (1)	The answer shows some understanding or knowledge of the topic, but there are significant errors or missing elements. Although not completely incorrect, the answer is not sufficiently correct or complete to be considered certain or adequate.
Correct (2)	The answer is completely accurate and shows a solid and precise understanding of the subject. All major components are addressed in a thorough and accurate manner.

All data obtained were recorded in an Excel spreadsheet (Excel version 16; Microsoft Corp). STATA statistical software program (STATA version BE 14; StataCorp) was used to analyze the data. The relative frequency (n) and absolute percentage (%) of the generated answers, categorized according to the gradings given by the experts (0 = incorrect; 1 = incomplete or partially correct; 2 = correct). The consistency of each expert and level of agreement between experts’ gradings was assessed for the entire set of answers generated by ChatGPT-4.

To evaluate the performance of ChatGPT-4 in generating answers in implant-supported prostheses, reliability and repeatability were examined for each prompt used (general or specific). Reliability was calculated as the proportion of questions yielding an answer with a grade of 2 (correct), along with its 95% confidence interval (Wald binomial method). This calculation was performed for the total set of answers as well as for each individual question. The difference in reliability between the general and specific prompts was examined using the Chi-square test, and Cramer’s V effect size was calculated. Repeatability was examined using concordance analysis weighted by ordinal categories and multiple repetitions with 95% confidence intervals (percent agreement, Brennan and Prediger coefficient, Conger generalized Cohen kappa, Fleiss kappa, Gwet AC, and Krippendorff alpha).According to the benchmark scale proposed by Gwet [32], the estimated coefficients were classified as follows: < 0.0 Poor, 0.0–0.2, Slight, 0.2–0.4 Fair, 0.4–0.6 Moderate, 0.6–0.8 Substantial, 0.8–1.0 Almost Perfect. The difference in repeatability between the general and specific prompts was analyzed by examining the overlap of the 95% confidence intervals for the coefficients.

## Results

The reliability distribution of expert grading of the 1800 implant-supported prostheses answers generated by ChatGPT for the general prompt and the specific prompt are shown in Table 4.

**Table 4:** Distribution of expert gradings for ChatGPT answers with the general prompt.

Question	Prompt 1						Prompt 2						p-value (General vs specific Prompt)
	Incorrect		Partially Correct or Incomplete		Correct		Incorrect		Partially Correct or Incomplete		Correct		
	n	%	n	%	n	%	n	%	n	%	n	%	
Q1	0	0.00	0	0.00	30	100.00	0	0.00	0	0.00	30	100.00	0.820
Q2	0	0.00	30	100.00	0	0.00	0	0.00	28	93.33	2	6.67	0.809
Q3	0	0.00	0	0.00	30	100.00	0	0.00	0	0.00	30	100.00	0.842
Q4	0	0.00	0	0.00	30	100.00	0	0.00	1	3.33	29	96.67	0.842
Q5	0	0.00	0	0.00	30	100.00	0	0.00	0	0.00	30	100.00	0.247
Q6	19	63.33	4	13.33	7	23.33	0	0.00	0	0.00	30	100.00	0.881
Q7	0	0.00	0	0.00	30	100.00	0	0.00	0	0.00	30	100.00	0.920
Q8	30	100.00	0	0.00	0	0.00	30	100.00	0	0.00	0	0.00	0.524
Q9	0	0.00	0	0.00	30	100.00	0	0.00	0	0.00	30	100.00	0.728
Q10	0	0.00	0	0.00	30	100.00	0	0.00	0	0.00	30	100.00	0.848
Q11	0	0.00	30	100.00	0	0.00	0	0.00	0	0.00	30	100.00	0.474
Q12	0	0.00	0	0.00	30	100.00	0	0.00	0	0.00	30	100.00	0.842
Q13	20	66.67	6	20.00	4	13.33	1	3.33	0	0.00	29	96.67	0.368
Q14	20	66.67	8	26.67	2	6.67	4	13.33	26	86.67	0	0.00	0.686
Q15	0	0.00	0	0.00	30	100.00	0	0.00	0	0.00	30	100.00	0.690
Q16	0	0.00	0	0.00	30	100.00	0	0.00	8	26.67	22	73.33	0.744
Q17	0	0.00	0	0.00	30	100.00	0	0.00	0	0.00	30	100.00	0.685
Q18	0	0.00	0	0.00	30	100.00	0	0.00	19	63.33	11	36.67	0.826
Q19	0	0.00	0	0.00	30	100.00	0	0.00	0	0.00	30	100.00	0.473
Q20	0	0.00	0	0.00	30	100.00	0	0.00	0	0.00	30	100.00	0.670
Q21	1	3.33	29	96.67	0	0.00	0	0.00	28	93.33	2	6.67	0.476
Q22	0	0.00	0	0.00	30	100.00	0	0.00	0	0.00	30	100.00	0.670
Q23	3	10.00	27	90.00	0	0.00	18	60.00	4	13.33	8	26.67	0.323
Q24	0	0.00	0	0.00	30	100.00	0	0.00	0	0.00	30	100.00	0.606
Q25	0	0.00	0	0.00	30	100.00	1	3.33	0	0.00	29	96.67	0.329
Q26	0	0.00	0	0.00	30	100.00	0	0.00	0	0.00	30	100.00	0.601
Q27	13	43.33	10	33.33	7	23.33	1	3.33	8	26.67	21	70.00	0.648
Q28	0	0.00	11	36.67	19	63.33	10	33.33	10	33.33	10	33.33	0.375
Q29	0	0.00	0	0.00	30	100.00	0	0.00	0	0.00	30	100.00	0.613
Q30	0	0.00	1	3.33	29	96.67	0	0.00	0	0.00	30	100.00	0.368

Regarding the consistency of the reviewers’ gradings, expert 1 achieved an agreement percentage of 94.98% in grading the 1,800 answers generated by ChatGPT, with a Gwet’s AC1 of 91.94%. Similarly, expert 2 achieved an agreement percentage of 95.43%, with a Gwet’s AC1 of 92.57% for the same set of answers. Expert agreement was observed in 1,688 (93.78%) of the 1,800 answers generated by ChatGPT-4. In only 112 of the 1,800 responses did the experts disagree, requiring the intervention of Expert 3 to resolve the discrepancy.

**Fig 1:**
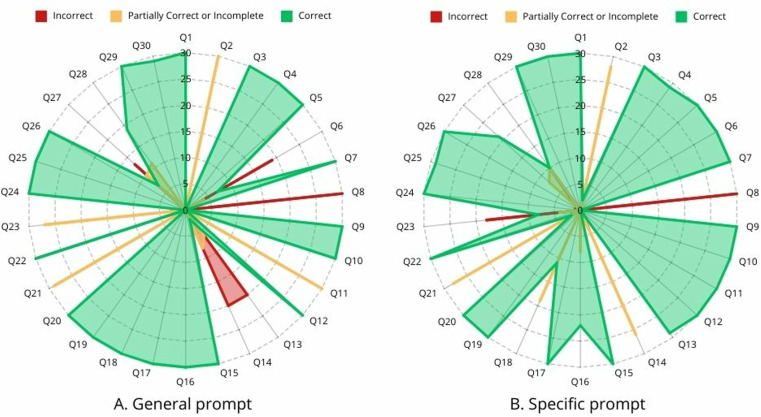
Number of incorrect, partially correct, or incomplete and correct answers obtained from ChatGPT for the 30 questions asked, according to the type of prompt used.

The percentage of reliable answers ranged from 0 to 100% depending on the question and prompt used. Of the 30 questions posed to ChatGPT using the general prompt, 19 received correct answers in all 30 repetitions (i.e., 100% reliability for those questions). Conversely, 5 questions did not receive any correct answers across the 30 repetitions (i.e., 0% reliability for those questions). Meanwhile, using the specific prompt, 18 questions achieved 100% reliability, and only 2 questions had 0% reliability (Fig 1).

Overall, the set of questions asked with the general prompt showed a reliability of 70.89% with a 95% confidence interval ranging from 67.84% to 73.76%. The specific prompt showed a reliability of 78.8% with a 95% confidence interval from 75.29% to 80.69%. Thus, the reliability of the specific prompt was significantly higher than the reliability of the general prompt (p < 0.001) (p < 0.001; Cramer’s V effect size = 0.083). However, when analysing each question separately (general prompt vs. specific prompt), no statistically significant differences were found (Table 4).

**Fig 2:**
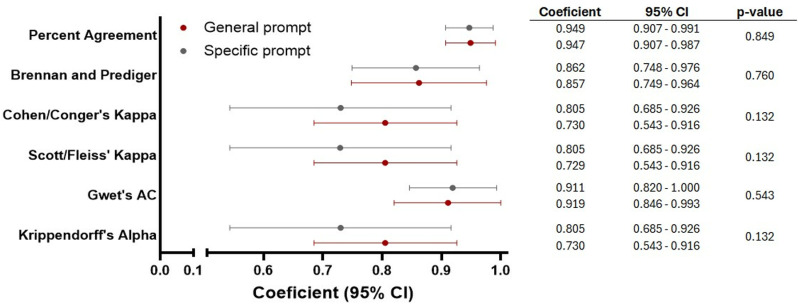
Repeatability (degree of agreement) of answers generated by ChatGPT using a general prompt and a specific prompt.

The repeatability of the experts’ gradings of the generated answers ranged from moderate to almost perfect for the general prompt (Table 5) and from substantial to almost perfect for the specific prompt (Table 6). The pronounced overlap of the 95% confidence intervals for the different coefficients indicates a lack of significant differences in the repeatability of answers between general and specific prompts (Fig 2).

**Table 5:** Evaluation of repeatability, based on expert grading, for 30 repetitions of 30 questions generated by ChatGPT with the general prompt.

Methods	Coeficient	SE	95% CI Range		Benchmark scale
Percent Agreement	0.949	0.021	0.907	0.991	Almost Perfect
Brennan and Prediger	0.862	0.056	0.748	0.976	Substantial
Cohen/Conger’s Kappa	0.805	0.059	0.685	0.926	Substantial
Scott/Fleiss’ Kappa	0.805	0.059	0.685	0.926	Substantial
Gwet’s AC	0.911	0.045	0.820	1.000	Almost Perfect
Krippendorff’s Alpha	0.805	0.059	0.685	0.926	Substantial

Benchmark scale: Poor <0.0, Slight 0.0–0.2, Fair 0.2–0.4, Moderate 0.4–0.6, Substantial 0.6–0.8, and Almost Perfect 0.8–1.0. CI, confidence interval; SE, Standard error.

**Table 6:** Evaluation of repeatability, based on expert grading, for 30 repetitions of 30 questions generated by ChatGPT with the specific prompt.

Methods	Coeficient	SE	95% CI Range		Benchmark scale
Percent Agreement	0.947	0.019	0.907	0.987	Almost Perfect
Brennan and Prediger	0.857	0.052	0.749	0.964	Substantial
Cohen/Conger’s Kappa	0.730	0.091	0.543	0.916	Moderate
Scott/Fleiss’ Kappa	0.729	0.091	0.543	0.916	Moderate
Gwet’s AC	0.919	0.036	0.846	0.993	Almost Perfect
Krippendorff’s Alpha	0.730	0.091	0.543	0.916	Moderate

Benchmark scale: Poor <0.0, Slight 0.0–0.2, Fair 0.2–0.4, Moderate 0.4–0.6, Substantial 0.6–0.8, and Almost Perfect 0.8–1.0. CI, confidence interval; SE, Standard error.

## Discussion

ChatGPT performance evaluation aimed to analyse the generation of answers in the field of implant-supported prostheses. For this purpose, questions were formulated using two types of prompts, and the generated answers were graded by experts to measure their reliability and repeatability. The research hypothesis that the implant-supported prostheses answers provided by ChatGPT-4 would not exhibit reliability and repeatability was partially rejected as the answers showed limited levels of reliability and repeatability, although better performance was observed when using the specific prompt.

As the accessibility of AI has shown a significant increase, the performance of ChatGPT in generating answers to dental questions needs to be evaluated. The results of this study show a reliability of 70.89% with the general prompt, and a reliability of 78.8% with the specific prompt. These results were higher than those observed in a study that analysed the performance of ChatGPT on questions about implant-supported and implant-retained prostheses, where a mean reliability of 25.6% was observed [17]. However, different performance rates have been reported in other dental specialties. Similar results to this study have been observed in Dental Surgery. It has been reported a mean reliability of 71.7% for Dental Surgery answers, with the proportion of correct answers varying between 0 and 100% [10] and values of 3.94, 3.85 and 3.96 over 4 for answers related to anatomical landmarks, oral and maxillofacial pathologies and radiographic features of the pathologies, respectively [6]. Nevertheless, better values were found for patient questions (4.62 out of 5) than for technical questions (3.1 out of 5) [11]. Furthermore, in Periodontology, several studies evaluating the performance of ChatGPT on patient questions found that the quality of most answers was rated as “good” based on the DISCERN instrument [12], while the accuracy and completeness for periodontal questions were 5.5 out of 6 and 2.3 out of 3, respectively [13]. These differences between the studies might be related to the specific dental specialty, as ChatGPT retrieves information from different Internet sources [1] whose origin is unknown [10]. In addition to comparing the performance of ChatGPT in different dental specialties, it is also important to evaluate its performance in relation to other LLMs. In this regard, several studies [33,34] have highlighted ChatGPT-4 as the best performing model. However, another study in the field of orthodontics [35] found no significant differences between the models, with Microsoft Bing Chat ranking highest, followed by ChatGPT-4, Google Bard and ChatGPT-3.5. However, the different versions of the models used, the data collection periods, and the different specialties may have influenced the variability of results between studies.

Repeatability is a factor to consider as ChatGPT might not always give the same answer to the same question [8]. Furthermore, ChatGPT might randomly give seemingly correct answers mixed with incorrect answers [25]. In this study, from moderate to almost perfect repeatability range was obtained for the general prompt, and from substantial to almost perfect repeatability for the specific prompts. However, the number of studies investigating the repeatability of answers in ChatGPT is limited. Previous research has shown that the repeatability for generating dichotomous endodontic answers was 85.44% [8], and was similar between ChatGPT.3–5, Google Bard and Bing [9]. However, the repeatability of ChatGPT-4 varied depending on the dental specialty analyzed. In Oral Surgery, repeatability was observed with moderate to almost perfect ranges [10], while in Prosthodontics substantial to moderate ranges were found [17].

Regarding the prompts used, it was observed that the specific prompt had a better performance than the general prompt, and this difference was statistically significant in the reliability of the generated answers. These results are in line with previous studies that emphasise the importance of careful prompt design to ensure high quality outputs [36].

Therefore, the way a prompt is designed, known as prompt engineering, should be considered [37], as it is a key factor in optimising model performance. Prompt engineering involves formulating effective instructions that efficiently guide models to generate the desired response. [38,39].

Thus, the observed results could therefore be attributed to the additional clarity given to ChatGPT about its role, the target audience, and the instruction to respond in a precise and direct manner, without digressions or creative answers. This increased guidance may have facilitated the generation of more accurate and relevant answers. Therefore, the use of a specific prompt by the professional could be recommended to optimise the performance of ChatGPT. In the field of dentistry, most studies [8,9,17] have used general prompts to formulate the question, and only a few studies specifically framed the question in ChatGPT [5,10] further research is therefore needed to determine the impact of different types of prompts on the quality of answers generated by ChatGPT to to determine their precise effect and optimise their design to improve the model’s performance.

According to the results obtained, the use of ChatGPT to generate answers in implant-supported and implant-retained prostheses is promising. However, given the level of reliability and repeatability, as well as the unreliability observed, it needs to be carried out under the supervision of a professional. In addition, it would be recommended to use a specific prompt that provides more accurate answers. Further research is needed to analyze the performance of ChatGPT in implant-supported prostheses, as well as the analysis of different prompts to obtain the most accurate answers.

An advantage of this study is the number of answers analyzed, a total of 1800. This large dataset provides a solid basis for evaluating the performance of ChatGPT in implant-supported prostheses, allowing more robust and representative conclusions to be drawn about its reliability and repeatability. In addition, this study compares the use of different prompts, thus contributing to the understanding of how question wording might affect the performance of ChatGPT. To ensure the high quality of the questions entered into ChatGPT, they were designed by the researchers based on *The Proceedings of the Seventh ITI Consensus Conference* [29]. These questions were evaluated by experts with over 15 years of experience in the field using a 3-point Likert scale (0 = disagree, 1 = neutral, 2 = agree). The 30 highest-scoring questions were selected for the study, reducing potential biases related to the questions. Regarding the quality of answer grading, the experts graded the 1,800 answers generated by ChatGPT, both with a high consistency. The level of agreement between expert 1 and expert 2 was high. Additionally, in case of discrepancies, expert 3 intervened to resolve the differences. This helped to minimise potential biases in the grading process.

One of the main limitations of this study was that only technical questions were analysed, which may not fully represent the variety of questions encountered in clinical practice. The performance of ChatGPT may vary depending on the complexity and context of the questions, particularly in real-world clinical scenarios or patient-generated queries. Future studies should evaluate its reliability in these contexts.

Future research should investigate its reliability in these contexts, as well as its potential impact on clinical decision making. In addition, further studies should evaluate its performance with patient-generated questions and analyse the influence of different prompt design strategies to optimise response reliability. Moreover, comparing ChatGPT’s performance with other AI models would provide valuable insights into its relative strengths and weaknesses in implant prosthodontics. Expanding the scope of research in these areas would provide a more comprehensive understanding of the capabilities and limitations of ChatGPT in implant prosthodontics.

## Conclusions

ChatGPT showed promising reliability and repeatability in generating answers in implant-supported and implant-retained prostheses. Better results were obtained when using a specific prompt compared to a general prompt. However, the results suggest that ChatGPT should always be used under the supervision of a professional who can identify and manage limitations.

## Supporting information

S1 Appendix STAGER checklist. (PDF)

S2 Appendix TRIPOD checklist (PDF)
