# Supplementary material for: Evaluating the influence of prompt formulation on the reliability and repeatability of ChatGPT in implant-supported prostheses
Source: PLoS One. 2025 May 30;20(5):e0323086. doi: 10.1371/journal.pone.0323086 (PMC12124515; doi:10.1371/journal.pone.0323086)
Supplement: S1 Appendix — (PDF) [file pone.0323086.s001.pdf]

STAGER checklist: Standardized testing and assessment guidelines for evaluating generative artificial intelligence reliability

Section/Topic	Item	Recommendation	page
**Title**	1	Identify the report as an article related to the research that evaluates generative AI's applicability in medicine.	1
**Abstract**	2	State the purpose of the research, the generative AI model used and its version, the source of the questions, methods, results, and conclusions.	2
**Introduction**			
Justification	3	Review existing relevant information and explain the background of the study.	3-4
Objectives	4	State specific objectives, including the generative AI model used and its version, the training set used for generative AI, the source of the questions, the nature of research, and the	3-4
**Methods**			
Question Collection	5	Select the professional questions from guidelines, official examination question banks, and high-frequency issues found via search engines like Google, or drafted by experts, ensuring that the questions cover specific subfields of medicine.	5
	6	Ensure the questions are representative in terms of difficulty, type, and professionalism.	5-6
	7	Describe how the questions were collected, the number of questions, whether the questions were pre-screened, the conditions of the screening, the modality of the input as well as	5-10
Agent	8	Record the model used, the version of the generative AI, and customized parameters such as temperature parameter, if applicable. State the strengths and weaknesses of the current version used and the rationale for assessing it.	6
	9	If intend to report them as a functional series, it is recommended to report the relationship between model versions (e.g., whether it is a simple upgrade; and if not, it is recommended to report the horizontal comparison results).	NA
Questioning	10	Use a consistent prompt with identically formatted patterns and provide the full prompt in the article.	6-10, 18
	11	Ask the same question multiple times and record each response.	6
	12	Indicate whether the question is open-ended or multiple-choice.	5
	13	Initiate a new chat for each question.	6
	14	Record the data the responses were collected.	6
Accuracy	15	Describe any methods employed for scoring accuracy when dealing with subjective questions.	5-10
	16	Compare with reference answers, record the number of correct responses to each question, and calculate the rate of correct answers if you asked objective questions.	11-12
Integrity	17	Describe any methods used to assess the integrity between responses.	11-12
Readability	18	Describe any methods used to assess the readability of	11-12

Reviewers	19	Clarify the composition of reviewers and the rationale for this composition, which is recommended to be more than two experts from varied fields like medicine, artificial intelligence, and interdisciplinary areas, along with stakeholders from ethics, sociology, and user groups.	11
	20	Pay special attention to assessing the implementability of	5-10
	21	Evaluate the consistency across responses to the same question to assess whether the generative AI can steadily provide consistent responses.	13-16
	22	Assess the consistency and reliability of reviewer ratings, avoiding significant differences in the subjective scores among	13-16
**Results**			
Results Selection	23	Describe the results of the search process, from the number of questions collected to the final results, ideally using a flow	13-16
Study Characteristics	24	State all studies included in the analysis and detail their characteristics.	13-16
Results of Individual Studies	25	Present results for accuracy, completeness, and readability for each study, recommending the use of tables or charts for	13-16
Results of Syntheses	26	Present results of all statistical syntheses conducted, and results of analyses conducted to explore possible causes of heterogeneity among study results.	13-16
**Discussion**			
Interpretation	27	Analyze the results according to the study objectives.	16-20
Strengths and Limitations	28	Describe the advantages of the research.	18-19
	29	Explore constraints of the research, acknowledging possible origins of partiality or inaccuracy.	20
	30	Engage in rational discussion and reject exaggeration.	16-20
Conclusion	31	Provide a condensed conclusion that summarizes the study's main findings, reiterates its importance, and indicates directions or recommendations for future research.	20
**Other Information**			
Funding and Sponsorship	32	Provide the origin of financial support and the function of the sponsors for the current investigation, as well as for the initial research if relevant to the foundation of this article.	21
Abbreviations: AI, artificial intelligence			
